# Individual Sea Urchin Coelomocytes Undergo Somatic Immune Gene Diversification

**DOI:** 10.3389/fimmu.2019.01298

**Published:** 2019-06-06

**Authors:** Matan Oren, Benyamin Rosental, Teresa S. Hawley, Gi-Young Kim, Jacob Agronin, Caroline R. Reynolds, Leon Grayfer, L. Courtney Smith

**Affiliations:** ^1^Department of Molecular Biology, Ariel University, Ariel, Israel; ^2^Department of Biological Sciences, The George Washington University, Washington, DC, United States; ^3^French Associates' Institute for Agriculture and Biotechnology of Drylands, Jacob Blaustein Institutes for Desert Research, Ben-Gurion University of the Negev, Be'er Sheva, Israel; ^4^Institute for Stem Cell Biology and Regenerative Medicine, Stanford University School of Medicine, Stanford, CA, United States; ^5^Department of Pathology, Hopkins Marine Station, Stanford University, Pacific Grove, CA, United States; ^6^Flow Cytometry Section, Research Technologies Branch, National Institute of Allergy and Infectious Diseases, National Institutes of Health, Bethesda, MD, United States; ^7^Department of Marine Life Sciences, Jeju National University, Jeju City, South Korea

**Keywords:** somatic gene diversification, *SpTrf*, *Sp185/333*, single cell, immune system evolution, sea urchin, whole genome amplification (WGA), somatic recombination

## Abstract

The adaptive immune response in jawed vertebrates is marked by the ability to diversify somatically specific immune receptor genes. Somatic recombination and hypermutation of gene segments are used to generate extensive repertoires of T and B cell receptors. In contrast, jawless vertebrates utilize a distinct diversification system based on copy choice to assemble their variable lymphocyte receptors. To date, very little evidence for somatic immune gene diversification has been reported in invertebrate species. Here we show that the *SpTransformer* (*SpTrf* ; formerly *Sp185/333*) immune effector gene family members from individual coelomocytes from purple sea urchins undergo somatic diversification by means of gene deletions, duplications, and acquisitions of single nucleotide polymorphisms. While sperm cells from an individual sea urchin have identical *SpTrf* gene repertoires, single cells from two distinct coelomocyte subpopulations from the same sea urchin exhibit significant variation in the *SpTrf* gene repertoires. Moreover, the highly diverse gene sequences derived from single coelomocytes are all in-frame, suggesting that an unknown mechanism(s) driving these somatic changes involve stringent selection or correction processes for expression of productive *SpTrf* transcripts. Together, our findings infer somatic immune gene diversification strategy in an invertebrate.

## Introduction

**Figure 1:**
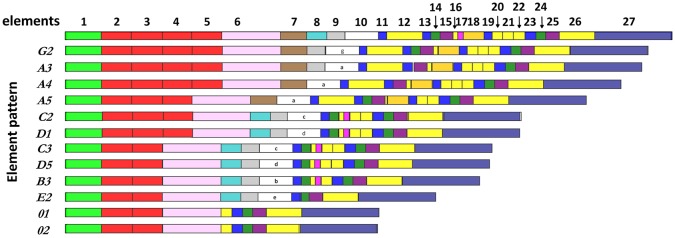
The *SpTrf* genes have a variety of element patterns. Element patterns identified for the genes amplified and sequenced in this study are shown. Elements are presented as rectangles of different colors. Element 10, which has very diverse sequence among genes, defines the gene name (4). This figure is based on data from Buckley and Smith (8).

The *Transformer* (*Trf* , formerly known as *185/333*) genes encode a family of highly diverse immune effector proteins in sea urchins (1–3). All *Trf* genes consist of two exons with the first exon encoding the leader and the second exon encoding the mature protein. The second exon has a mosaic structure composed of blocks of conserved sequences termed elements, that are either present or absent in different members of the gene family, creating defined element patterns (Figure 1) that are the major source of diversity in gene sequence and size (4, 5). In a screen of a genome library of the California purple sea urchin, *Strongylocentrotus purpuratus*, 15 *SpTrf* gene family members were identified in three clusters of tightly linked genes, including duplicated genes with 2–6 almost identical copies (6, 7). Previous estimates of the gene family size suggested ~50 members (8). However, the number of genes and their composition significantly varies in the sea urchin population (9). Accordingly, different sea urchin genotypes express different subsets of *SpTrf* transcripts (8, 10) and protein repertoires (11). The SpTrf protein variants maintain an overall conserved structure of a glycine-rich region with a protein multimerization motif followed by a histidine-rich region, and a C-terminal region (4, 10, 12). The protein sequences have no predictable secondary structure (1, 3). In accordance, a recombinant SpTrf protein, rSpTrf-E1, is an intrinsically disordered protein, which transforms to α helical upon binding to different pathogens and cell membrane-associated motifs (12–14). Native SpTrf proteins opsonize bacteria, which augments phagocytosis and retards growth for some species of bacteria (12, 15). The *SpTrf* immune effector arm is highly responsive to immune challenge. Upon injection with pathogen associated molecular patterns (PAMPs) (8, 10) or *Vibrio diazotrophicus* (15), both *SpTrf* transcripts and SpTrf protein levels increase dramatically (10, 11, 16, 17). At the same time, the concentration of SpTrf-positive (SpTrf^+^) cells in the coelomic fluid (CF) increase (18). The *SpTrf* transcript repertoire shows a broad range of transcript sizes in coelomocytes prior to immune challenge, which changes toward a single size in response to immune challenge (10), suggesting functional specificity of individual *SpTrf* variants to particular targets.

Analysis of the *SpTrf* gene repertoire in individual coelomocytes from purple sea urchins shows that each cell contains *SpTrf* transcripts of a single sequence, suggesting expression of a single *SpTrf* gene per cell (19). As a follow-up to this finding, we tested the hypothesis that this restricted expression is the outcome of modifications in the genomic structure and gene content of the *SpTrf* gene family in individual coelomocytes. Our results suggest several types of *SpTrf* gene diversification within subpopulations of sea urchin coelomocytes including gene deletion, duplication, and single nucleotide polymorphism. We hypothesize that this diversification strategy is unique and beneficial for the survival of sea urchins because it broadens the array of immune effector proteins that are produced and bind a wider variety of pathogens and initiate mechanisms of immune protection.

## Results

### The Proportions of *SpTrf*^+^ Cells Increase in Response to Immune Challenge

**Figure 2:**
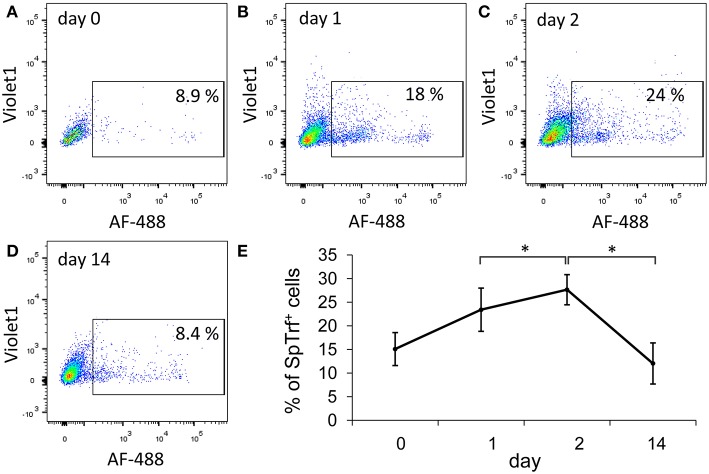
The percentages of *SpTrf* ^+^ small phagocytes in the coelomic fluid increase in response to *Vibrio* challenge. Three adult sea urchins were injected with heat-killed *Vibrio diazotrophicus*. Coelomic fluid (CF) was collected prior to injection (day 0) and after injection on days 1, 2, and 14. **(A–D)** Cells were incubated with a mix of three anti-SpTrf (formerly anti-Sp185/333) primary antibodies followed by incubation with a secondary antibody labeled with Alexa Fluor 488 (AF-488) to identified SpTrf^+^ cells. Nuclear staining with propidium iodine was used to exclude dead cells. Results for sea urchin 1 are shown. Percentages of live SpTrf^+^ coelomocytes from animal 1 at all time points are shown within the rectangular gate. SpTrf^+^ coelomocytes increase to the highest recorded level on day 2 post challenge and return to the basal level by day 14. **(E)** The mean of the percentages of SpTrf^+^ coelomocytes for the three individual animals at different time points are shown. Differences in the percentages of SpTrf^+^ cells over time for the individual animals were significant by Anova two factor analysis (*p* < 0.05). Additionally, the day 2 time point was analyzed compared to days 1 and 14 time points using Anova single factor (*indicates *p* < 0.05). Vertical bars indicate standard errors.

**Figure 3:**
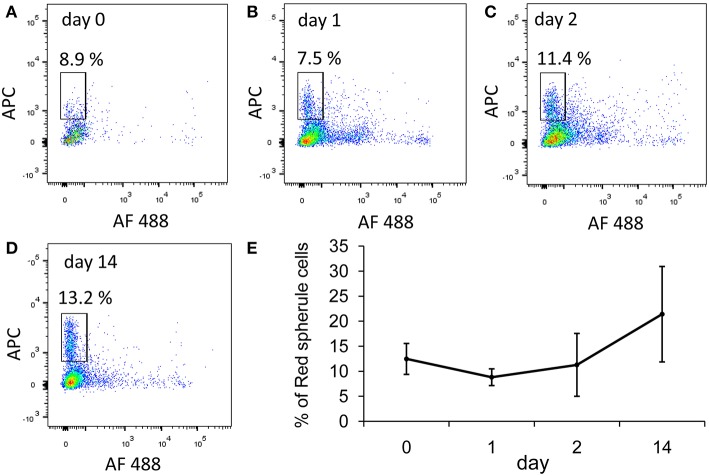
The percentages of red spherule cells in the purple sea urchin coelomic fluid does not change in response to *Vibrio* challenge. Three adult sea urchins were injected with the heat-killed marine bacterial species, *Vibrio diazotrophicus*. Coelomic fluid was collected before injection (day 0) and post injection at days 1, 2, and 14. Cells were labeled with propidium iodine to exclude dead cells. Red spherule cells were gated by their far red auto-fluorescence (APC channel). **(A–D)** Percentages of live red spherule cells from animal #1 at all time points (within the rectangular gate) in the coelomic fluid over 14 days post challenge. **(E)** Percentages of red spherule cells in the total live coelomocyte population of the three animals tested. Differences in the red spherule cell percentages over time were not significant by Anova two factor analysis. Standard deviation is indicated by vertical bars.

**Figure 4:**
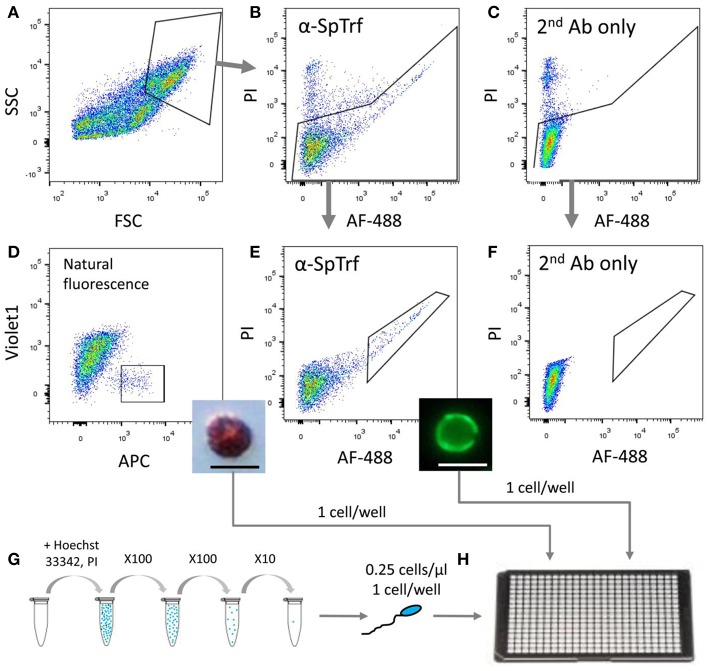
Sorting and isolation of single cells from two coelomocyte sub-populations and from sperm for WGA. Small phagocytes that express SpTrf on their surface and red spherule cells were sorted from animals 1–3 pre-challenge on day 0 as well as on days 1 and 2 post-challenge with *Vibrio diazotrophicus*. Sperm cells were collected directly from the gonopores of the same sea urchins. **(A)** Coelomocytes were gated from debris based on forward scatter (FSC) and side scatter (SSC). **(B)** Live cells were gated for propidium iodide (PI) exclusion and for surface staining of anti-SpTrf and secondary antibody conjugated with Alexa Fluor 488 (AF-488). **(C)** Live cells incubated with the secondary antibody alone did not show AF-488 staining. **(D)** Red spherule cells were gated based on their natural far-red auto-fluorescence (allophycocyanin channel; APC). Single cells with high auto-fluorescence were sorted and observed post-sorting by light microscopy (inset). **(E)** Live cells with high surface SpTrf protein levels were gated based on the AF-488 fluorescence of the secondary antibody. Single cells were sorted and observed post-sorting by fluorescence microscopy (inset). **(F)** No cells were recorded in the same gate as in **(E)** for the sample incubated with the secondary antibody only. This demonstrated that all cells within the gate in **(E)** had SpTrf on the surface and were likely small phagocytes. **(G)** Sperm cells were obtained by electric shock stimulation (16–20 mA), diluted to 1×10^5^ cells/ml, stained with Hoechst 33342 and serially diluted to 0.25 cells/μl. **(H)** Single cells of each type were isolated either by FACs or manually into 4 μl of 3.3X PBS in a 384-well plate, observed under epifluorescent microscope, and subjected to WGA using the multiple displacement amplification with the REPLI-g single-cell kit (Qiagen). The scale bars in the insert figures in **(D**,**E)** are 10 μm.

We focused on two immune-relevant populations of sea urchin coelomocytes, (i) cells that expressed SpTrf proteins on their surface that likely consisted of small phagocytes (18). These cells could be isolated by fluorescence activated cell sorting (FACS) based on anti-SpTrf antibodies. (ii) SpTrf-negative red spherule cells were isolated by FACS based on their far-red auto-fluorescence. Red spherule cells contain the pigment echinochrome A in cytoplasmic vesicles and shows antimicrobial activity upon exocytosis (20). To test whether immune challenge with heat-killed *Vibrio diazotrophicus*, a marine bacterial pathogen (21), induces a cellular immune response, we measured the relative concentrations of SpTrf^+^ coelomocytes and red spherule cells in the CF, before challenge as well as on days 1, 2, and 14 post-challenge (Figure 2). These time points were chosen based on previous studies showing increase in the SpTrf transcripts levels at 1–2 days post-challenge [e.g., Terwilliger et al. (10)]. As expected, the percentages of SpTrf^+^ cells within the coelomic fluid of the three tested sea urchins sharply increased within the first day, with the highest value measured at 2 days post challenge, followed by a decrease to the basal level after 14 days (Figure 2E). This pattern was in agreement with results reported previously (18, 19). Furthermore, at all time points there were wide variations in the intensity of the SpTrf protein expression among different SpTrf^+^ cells (Figures 2A–D, 4E). On the other hand, the percentages of the red spherule cell population in the CF in response to *V. diazotriphicus* showed no significant change over the same time period (Figure 3). These results reflected a typical immune response of sea urchins to the *Vibrio* pathogen and provided an experimental basis for additional investigation.

### Single Coelomocyte Genomes Contain Different *SpTrf* Gene Repertoires

**Figure 5:**
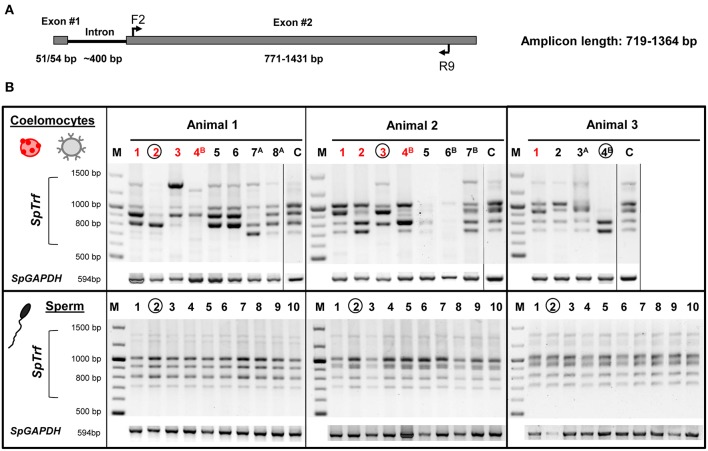
The *SpTrf* gene family profile is different among single coelomocytes. **(A)** The standard structure of an *SpTrf* gene shows the locations of F2 and R9 degenerate primers that were used to amplify most of the second exon. This figure is modified from (6). **(B)** *SpTrf* gene amplification profiles from single coelomocytes and single sperm from three sea urchins that were processed for WGA. The upper panel shows the amplicon patterns from individual coelomocytes from three animals. Both single red spherule cells (red numbers) and single small phagocytes (black numbers) show variable sizes of the *SpTrf* gene amplicons. Circled lane numbers indicate samples for which amplicons were sequenced and include one coelomocyte and one sperm from each animal. Superscript letters associated with lane numbers indicate the time after immune challenge with *Vibrio diazotrophicus*: ^A^, 1 day post challenge; ^B^, 2 days post challenge. The controls (C) for each of the three sea urchins show amplicons from ~10^6^ coelomocytes that were not processed for WGA. The lower panel shows the amplicon patterns of 10 single sperm cells from each animal. *SpGAPDH* is a single copy gene that was employed as the positive control for all samples to verify that genomic DNA after the WGA process would support PCR.

To determine whether individual sea urchin coelomocytes undergo changes in the numbers of their respective *SpTrf* gene family members, we employed FACS to sort and isolate single small phagocytes and single red spherule cells, which were confirmed by visual inspection (Figure 4). Cells from each cell population from three sea urchins were sorted at three time points relative to challenge (days 0, 1, and 2). Single sperm were isolated from the same three sea urchins using limiting dilution followed by visualization of sperm by live/dead staining with Hoechst and propidium iodide (see materials and methods). All single cells were processed directly for whole genome amplification (WGA) by the multiple displacement amplification method (22). Genomic DNA from 19 of 54 sorted coelomocytes that included 10 small phagocytes and 9 red spherule cells plus 30 of 36 isolated sperm cells were amplified successfully based on the following parameters: (i) WGA products were observed as smears of DNA fragments by gel electrophoresis with a majority of amplified fragments of ~20 kb (Figure S1), (ii) The control gene, *SpGAPDH* (genebank ref. XM_775023.4), was amplified successfully by PCR from all single cell WGA products (Figure 5B), and (iii) *SpGAPDH* amplicon sequences originated from the same genotype were identical or differ in only one nucleotide (Figure S2). The amplified genomes from single cells were used to characterize the *SpTrf* gene repertoires using degenerate primers (F2/R9; Table S1; Figure 5A). Because the second exon is highly variable in sequence and size (8), a series of different *SpTrf* gene amplicons was obtained for individual cells (Figure 5B). Amplicons from individual sperm cells from the three sea urchins consisted of six or seven bands and appeared identical for all sperm cells collected from individual animals, but differed slightly in number and length among the animals (Figure 5B, bottom panels). This suggested distinct gene repertoires in different sea urchin genotypes, which was in agreement with previous reports (4, 6, 8). In contrast, most single coelomocytes exhibited diverse amplicon patterns when compared within one animal or among animals (Figure 5B, top panels).

The SpTrf amplicon patterns from single coelomocytes differed from the patterns derived from the corresponding sperm cells, both in number and relative intensity. Most single coelomocyte samples were deficient for one or more amplicons of specific sizes, suggesting the absence of *SpTrf* gene(s) of specific sizes from their respective genomes. For example, small phagocyte #4^B^ from animal 3 had amplicons of ~0.7 and ~0.8 kb, whereas the sperm samples from animal 3 had at least four additional amplicons of larger sizes (Figure 5B). Amplification of genomic DNA isolated from ~10^6^ coelomocytes from each animal, resulted in a pattern which included all bands observed collectively in the individual coelomocytes (Figure 5B, lanes labeled C). Variations in amplicons among coelomocytes were observed for both red spherule cells and in the small phagocytes at all time points before and after the immunological challenge suggesting that both coelomocyte populations contained modified *SpTrf* gene repertoires.

### *SpTrf* Gene Sequences Vary Across Genotypes and Between Germline and Coelomocytes

**Figure 6:**
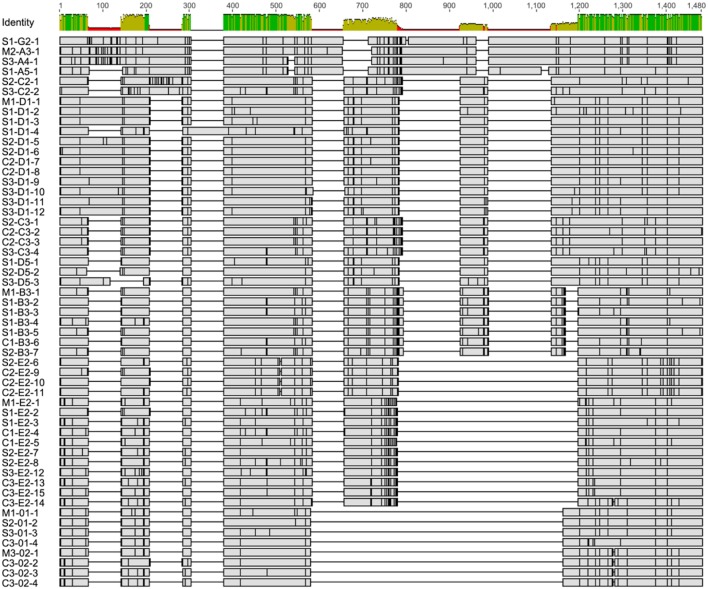
The alignment of single cell derived *SpTrf* gene amplicon sequences indicate different element patterns and SNPs. All sequences were aligned using Bioedit software (46). The initial alignment was done with ClustalW on the deduced amino acid sequences, which was reverted to nucleotide sequences and optimized manually. The sequence identity level (percent identity) for each position is indicated at the top. Horizontal gray rectangles show regions of matching nucleotides in which SNPs are indicated as vertical black lines. Gaps are shown as black horizontal lines and indicate missing elements. Sequence names indicate source based on sperm (S), coelomocytes (C), or both (M), followed by element pattern, and sequence version number. Detailed alignment is presented in Figure S4.

**Figure 7:**
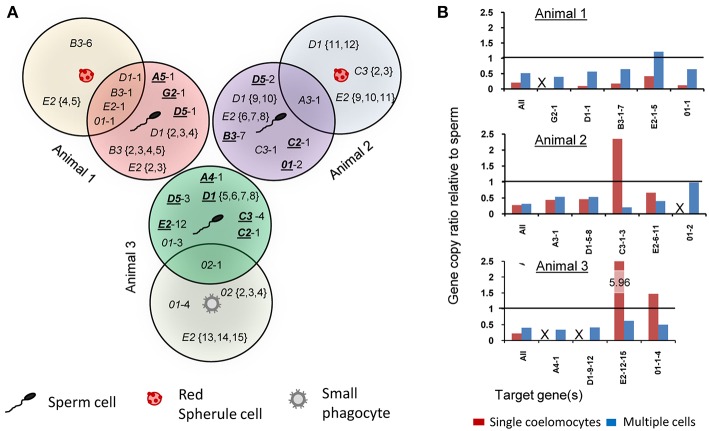
Single cell analysis indicates somatic gene deletion, duplication, and sequence diversification in the *SpTrf* gene family. Amplicons of one coelomocyte and one sperm from each animal (as indicated in Figure 5B) were cloned and sequenced. **(A)** The Venn diagram illustrates shared and unique gene amplicons (element pattern followed by a sequence variant number after the dash) from single coelomocytes and single sperm from three animals. Underlined element patterns indicate gene sequences that are unique to sperm and are not found in coelomocytes for each animal. **(B)** qPCR results using primers that amplify the second exon of all *SpTrf* genes plus primers designed for specific gene sequences are shown as ratios of coelomocyte amplicons relative to sperm amplicons for each animal. Ratios of gene copy number between multiple coelomocyte and multiple sperm samples that were not processed for WGA are indicated in blue. Ratios of gene copy number between single coelomocyte and single sperm from the same animal (same cells as in **A**) are shown in red. The horizontal line represents a ratio of 1 (identical gene copy numbers between coelomocyte and sperm). Samples in which primers did not generate amplicons by qPCR are indicated with an X. See Table S2 for details.

To verify that the single cell amplicons were *SpTrf* gene sequences and to compare sequences among coelomocytes and sperm, amplicons from one sperm and one coelomocyte from each of the three animals were cloned and sequenced. The amplicon sequences were compared and categorized based on published *SpTrf* gene sequence alignments and naming convention (8, 10), according to their element patterns (Figure 1) (8). Sequences were aligned automatically with subsequent editing manually to achieve an optimal alignment (Figure 6). Aligned amplicon sequences identified 12 different categories that differed in lengths and element patterns. All amplicon sizes that were observed by gel electrophoresis matched the lengths of the amplicon sequences (i.e., for each band size we found at least one matching sequence size). The element patterns from the three sea urchins were further sub-categorized according to variations in sequence that was based on single nucleotide polymorphisms (SNPs) resulting in 54 unique sequences of which 48 were identified more than once (~86% redundancy). The numbers of unique sequences were similar among animals (17–19 per animal), however, the number of sequence variants per element pattern ranged from 1 to 15 (Table S2). In accordance with previous results (1, 8), no shared *SpTrf* gene sequences were identified among animals (Figure 7A). When gene sequences were compared among cells from individual animals, one to four sequences were shared between the coelomocyte and sperm cell, while 15 to 17 were unique to either the coelomocyte or the sperm (Figure 7A). This included both unique element patterns and unique sequences (or sub-categories) of the same element pattern. Three to six unique element patterns identified in sperm were not identified in the coelomocyte for individual animals (Figure 7A, underlined).

To verify the *SpTrf* gene sequence diversity for the single cell samples and to estimate the *SpTrf* gene copy ratios between single coelomocytes and sperm, we performed qPCR on the WGA samples. We used four to five primer pairs that were specific for sequences of one or more *SpTrf* genes for a given animal, as well as primer pairs that targeted all gene sequences derived from each of the animals (Table S1). The gene amplicons were normalized to the single copy gene *SpGAPDH* and then gene copy ratios were calculated for single coelomocytes relative to single sperm for each animal. Most (14 of 17) of the amplified *SpTrf* gene amplicons were present at lower copy numbers in coelomocytes compared to sperm. The three exceptions of 17 cases showed the opposite with ratios above 1 (Figure 7B, red bars; Table S2). No amplification signal was detected for coelomocyte gene variants *G2*-1 (animal 1), *01*-2 (animal 2), and *A4*-1 and *D1*-9-12 (animal 3) (Figure 7B, indicated as “X”). These missing gene variants in the coelomocyte samples corresponded to missing bands in the *SpTrf* gene amplicon results (Figure 5). Because the WGA procedure may cause bias in gene representation, we used the same primers to amplify the *SpTrf* genes from genomic DNA isolated from multiple coelomocytes and multiple sperm (~10^6^ cells) from the same three animals, which was not processed for WGA prior to qPCR. In general, the predicted *SpTrf* gene copy number ratios for multiple coelomocytes compared to multiple sperm cells did not match those calculated for single cells from respective animals and was lower than 1 in all but two cases (Figure 7B; blue bars). Taken together, these results suggested modifications to the *SpTrf* gene family within individual coelomocytes giving rise to distinct subsets of *SpTrf* genes in individual cells.

### *SpTrf* Genes Maintain Open Reading Frames That Are Selected for Diversification

In spite of the prediction for gene diversification and local genomic instability in the *SpTrf* gene family, which has tightly linked members that are associated with multiple types of repeats (6, 7, 9), only a single pseudogene has been identified of 198 genes that have been sequenced (6, 8). In accordance with published *SpTrf* sequences, all single cell amplicon sequences from the three animals had full-length ORFs encoding 224 to 440 amino acids in the second exon without frameshifts or early stop codons (Figure S3). The lack of pseudogenes in this family suggests the existence of a regulatory mechanism for maintaining accurate reading frames in gene sequences during diversification (6, 7). A total of 2251 SNPs (varients) were identified of which 466 showed variation frequency above 0.25 (Figure S4). To elucidate whether *SpTrf* nucleotide sequence diversification effected the deduced SpTrf protein sequences within single coelomocytes, sequence diversity (or entropy) was calculated for sequences of the same length and element pattern from the single cells for each of the three animals. We note that because most element pattern sub-categories from the three genotypes contained only a few sequences, or even a single sequence, traditional dN:dS analyses could not be performed. The diversity ratios for different element patterns were 1.21 to 2.1 (Table 1) suggesting an overall high ratio of non-synonymous to synonymous nucleotide polymorphisms. Specific positions under positive selection were identified using the likelihood ratio test (LRT) based on the *SpTrf* sequence alignment. The distribution of SNPs was spread across the sequences with overall positive selection at 13 sites based on significant LRT results (*p* < 0.1) (Figure S5). Results did not indicate hypermutated regions in the sequences in accordance with (8).

**Table 1:** Sequence diversity ratios indicate diversifying selection for the *SpTrf* genes.

**Element pattern**	**ORF length (bp)**	**Diversity**		**nt/aa diversity ratio**
		**(nt)^a^**	**(aa)^b^**	
*A3*	1,312	0.0026	0.0048	1.85
*C2*	982	0.0332	0.0403	1.21
*D1*	976	0.0204	0.0313	1.53
*C3*	906	0.0087	0.0149	1.71
*D5*	901	0.0184	0.0313	1.7
*B3*	880	0.0271	0.0513	1.89
*E2*	766	0.0315	0.0451	1.43
*01*	676	0.0143	0.0216	1.51
*02*	673	0.0132	0.0277	2.1

a *Diversity of nucleotide sequence as determined by the equation; diversity = –freq (nt) × ln[freq(nt)]*.

b *Diversity of amino acid sequence as determined by the equation; diversity = –freq (aa) × ln[freq(aa)]*.

### *SpTrf* Genes of Different Element Patterns Cluster Into Separate Phylogenetic Clades

**Figure 8:**
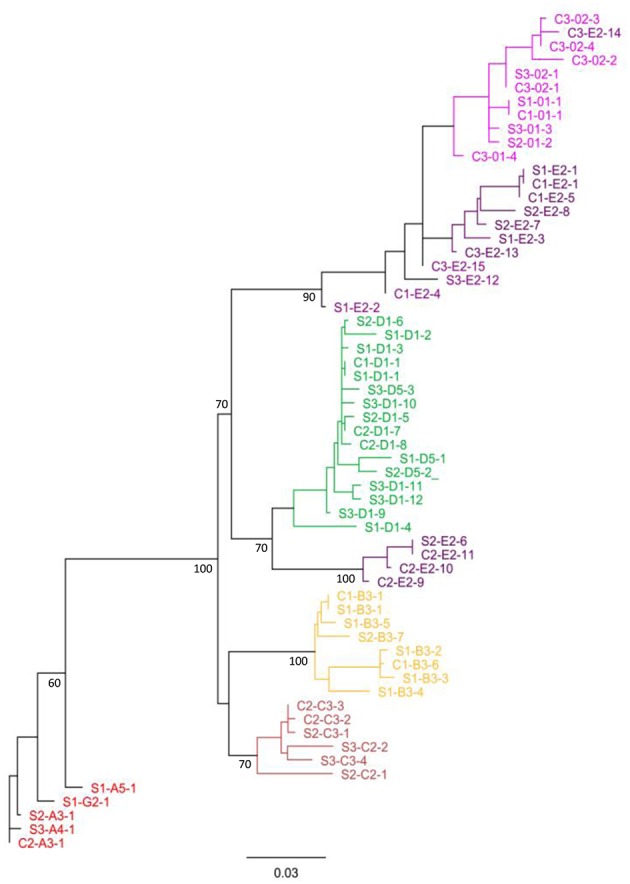
Single cell *SpTrf* gene sequences cluster according to element patterns. The phylogenetic relationships of *SpTrf* amplicon sequences containing most of the second exon were inferred using maximum parsimony, neighbor joining (results not shown), and maximum likelihood, all of which gave similar results. The tree shown here is based on the maximum likelihood phylogeny with PhyML according to Jukes-Cantor model with 1,000 bootstrap replicates. Bootstrap values for nodes present in >50% of trees are shown. Sequences from sperm (S) or coelomocyte (C) are indicated accordingly and accompanied with animal number (1, 2, or 3). The letter and number after the first dash indicate the element pattern based on previously published nomenclature (7, 8, 16). The last number after the second dash indicates the sequence sub-categories based on SNP variations within an element pattern. Colors indicate the gene element pattern according to Miller et al. (7) and Buckley and Smith (8).

To determine the phylogenetic relationships and evolutionary distances among the *SpTrf* genes from the single cells of the three animals, unrooted phylogenetic trees with calculated evolutionary distances were generated based on the *SpTrf* gene alignment (Figure 8). All phylogenetic trees that were produced using different phylogenetic approaches showed the same seven distinct clades. While the relationships among the clades were not always supported (i.e., had low bootstrap values), all clades were composed of a single element pattern (i.e., *E2, B3, C3*) or two very similar element patterns (i.e., *01/02, D1/D5, A/G*). One exception was the clade composed of both *E2* and *01/02* element patterns that included sequence C3-*E2*-14, which grouped within the *01/02* clade. The *E2* element pattern sequences were separated into two distinct clades suggesting two *E2* subtypes. One *E2* clade was paraphyletic to the *D1/D5* clade and consisted of sequences from a single genotype (animal #2), while the other clustered with the *01/02* clade and contained sequences from all three genotypes. A tendency toward sequence clustering according to genotype was recorded within clades, however no clear segregation was observed for sperm sequences (indicated with an S in the gene names) relative to those derived from coelomocytes (indicated with a C).

The structure of the tree and organization of gene clades indicated different genes in the basal vs. terminal clades suggesting a theoretical estimate of the evolutionary history of this family in the sea urchin population. It is noteworthy that the *A*/*G* genes with the longest sequences, were positioned basally, and the *0* genes of the shortest sequence, plus a subset of the *E* genes were positioned in a terminal clade (Figures 1, 8). The *C, D, B*, and a subset of the *E* genes, grouped in two sets of sister clades (*B* and *C, E*, and *D*), that were of intermediate length and positioned within central clades. Overall, the *E* pattern sequences were spread across longer evolutionary distances (Figure 8), suggesting higher diversification rates that may be relevant to the *E* pattern genes and their elevated expression levels relative to the other genes (10). Whether this organization suggests that the long genes are more ancestral whereas the short genes are more recently derived is speculative.

## Discussion

Over evolutionary time, immune systems in a wide range of organisms have acquired different gene diversification strategies to adapt to changing environments and to keep pace with the rapidly evolving pathogens with which they are associated. Invertebrates utilize a variety of molecular mechanisms to diversify immune gene families such as extensive alternative splicing of mRNAs encoding Down syndrome cell adhesion molecules in arthropods (23, 24), expanded gene families of variable domain-containing chitin-binding proteins in cephalochordates (25, 26), Toll-like receptor genes in sea urchins (27, 28), and somatic modifications of fibrinogen-related protein genes in fresh water snails (29, 30). However, the ability to rearrange, recombine, or assemble genomic segments to create immune gene diversity is regarded as a fundamental trait of adaptive immunity that is restricted to vertebrates (31, 32). Jawed vertebrates rely on the V(D)J somatic gene recombination system to generate extensive repertoires of T and B cell antigen receptors (33), which are further diversified by induced somatic hypermutations (34, 35). Jawless vertebrates employ an alternative somatic diversification system, which is based on a copy choice mechanism, to assemble leucine rich repeat modules into non-functional germline variable lymphocyte receptor (*VLR*) gene to generate VLR proteins with a wide capability of antigen binding (36, 37). Our results suggest that similar to the vertebrate adaptive immunity genes, the *SpTrf* immune genes are subjected to somatic modifications. In this case, however, new combinations of complete *SpTrf* genes are created in individual sea urchin cells. This is in contrast to the recombination process in the vertebrates where gene segments are recombined to form a single gene.

In agreement with earlier studies (6, 8, 9), complete *SpTrf* gene sequences are not shared among animals, which is consistent with an extremely rapid *SpTrf* diversification rate in the sea urchin population. Here we found that *SpTrf* gene diversification is not limited to the level of sea urchin populations but also occurs among single somatic cells (coelomocytes) within individual animals. Using single cell isolation, WGA and PCR-based approaches, we show that while the *SpTrf* gene repertoire in sea urchin sperm cells are identical within a genotype, individual coelomocytes have altered *SpTrf* gene repertoires with missing and duplicated gene amplicons as well as SNPs. It is noteworthy that no correlation was found between the immune challenge or between the coelomocyte subtypes (i.e., *SpTrf*^+^ cells or red spherule cells) and the variations in the *SpTrf* gene repertoires. This suggests that the *SpTrf* gene diversification process is initiated early in the differentiation of coelomocytes and may not be induced by exposure to immune elicitors.

We are aware that the procedure of WGA that was used to amplify single cell genomes in this study may be prone to biases based on non-uniformity of amplification (38) and %GC-bias (39). PCR biases may also be present, resulting in uneven amplification of different *SpTrf* gene amplicons or unsuccessful annealing of the primers to the genomic template. Therefore, it may be theoretically possible that some missing amplicon sizes in single coelomocytes as well as stronger amplification of others are the outcome of these technical problems. While it is unlikely that these biases would be restricted to coelomocytes and would not apply to the sperm samples, we nonetheless performed several controls to rule out this possibility. PCR-related biases were ruled out using multiple qPCRs with genotype-specific primer sets (5–6 per genotype). Similar amplicon copy number proportions were obtained between coelomocytes and their corresponding sperm cells, in addition to no amplification of the same *SpTrf* genes that were not amplified by the original F2/R9 primers. To rule out biases due to WGA with multiple displacement amplification (MDA), we performed qPCR with the same primers on unamplified genomic DNA isolates from multiple coelomocytes and multiple sperm of the same genotypes. Outcomes represent the cumulative effects of *SpTrf* sequence modifications in single cells of the same animal and demonstrate different *SpTrf* gene copy numbers in multiple coelomocytes compared with multiple sperm. Most gene copy number ratios (multiple coelomocytes vs. multiple sperm) were less than one, which is consistent with gene deletions from genomic DNA from coelomocytes.

We cannot be sure that we completely covered by cloning and sequencing the full gene content of the *SpTrf* gene family for each coelomocyte and sperm cell that was sampled. Nevertheless, we assume that we have obtained a good coverage of the gene family for the following reasons: (i) the total redundancy of *SpTrf* sequences was 86% suggesting that many of the genes were cloned and sequenced more than once, (ii) all amplicon sizes that appeared in the gel were identified in the actual sequences, and (iii) all *SpTrf* element patterns that were identified in a single coelomocyte were also identified in the sperm of the same genotype.

In addition to the differences in *SpTrf* gene copy numbers among sea urchin coelomocytes, we observed multiple SNPs in gene sequences derived from coelomocytes compared with those of the corresponding sperm cells as deduced from the *SpTrf* sequence alignment. SNPs are another basis for the high sequence diversity in the *SpTrf* gene family for different sea urchin genotypes. However, the above observations demonstrate the occurrence of this type of variation in single cells of the same genotype. Frequent SNPs caused by somatic single point mutations in the *SpTrf* genes resemble the activation-induced cytidine deaminase (AID) mutagenesis in the variable exon of the vertebrate immunoglobulin genes. Recent work by Fugman et al. (40) suggests five potential *S. purpuratus AID* homologs with mutagenesis activity that may be functional in the sea urchin immune system. While we cannot conclude at this stage that there is any relationship between one or more of these *AID* genes and the SNPs in the *SpTrf* sequences, this possibility should be considered.

Frequent mutations in the *SpTrf* gene sequences, had they occurred randomly, would have resulted in frameshifts and early stop codons. Hence, the general lack of pseudogenes in this family is highly unusual, especially for a gene family with shared sequences among members that are organized in tight clusters and are associated with a variety of repeats, which may drive genomic instability (7, 9). In this study, we obtained 54 unique *SpTrf* sequences of the second exon from single sea urchin cells of which all encode full length open reading frames. Therefore, we speculate that both somatic and germline diversification processes are highly regulated. While the mechanism(s) that regulate *SpTrf* sequence diversification are unknown, we have suggested that the GA short tandem repeats (STRs) surrounding all genes and the GAT STRs that are associated with large segmental duplications that include duplicated genes may be involved in the gene deletion and duplication events (6, 7, 9). Taken together, our findings suggest that the strategy of diversifying immune gene repertoires beyond genome constraints and through somatic DNA changes evolved long before the emergence of the vertebrate lineage. In light of our results, we propose that sea urchins use such strategies to increase the diversity of their *Trf* gene repertoires toward more potent immune responses.

## Materials and Methods

### Sea Urchins, Bacteria and Immune Challenge

Adult purple sea urchins, *Strongylocentrotus purpuratus*, were purchased from the Southern California Sea Urchin Co. (Corona del Mar CA) and maintained in a closed sea water system for 6–8 months to down-regulate their immune response to achieve immunoquiescence (41, 42). Immunoquiescent sea urchins were activated immunologically by one injection of 10^6^ heat-killed *Vibrio diazotrophicus* per ml of CF as described (19). CF volume was estimated according to Smith et al. (43). *V. diazotrophicus* (Gram negative marine bacteria; American Type Culture Collection #33466) was cultured at room temperature for 18 h in 5 ml of marine broth (3.44% marine broth, 0.3% yeast extract, 0.5% proteose peptone; Difco). Bacteria were heat-killed at 95**°**C for 15 min and washed with artificial CF [aCF; see (18)] before use.

### Coelomocyte Preparation

CF (500 μl) from adult sea urchins was withdrawn from the coelomic cavity using a 1 ml syringe with a 21 gauge needle and expelled directly into Ca-, Mg-free artificial sea water with 70 mM EDTA and 20 mM HEPES pH 7.4 [CMFSW-EH, see (44)] at a 1:1 ratio. Cells were filtered through gauze pads, pelleted for 5 min at 400 × *g* at 4°C and resuspended in staining medium (3.3X PBS with 20 mM HEPES pH = 7.4 and 1.5% fetal calf serum) according to Smith et al. (45). Cells were incubated with a mix of three polyclonal rabbit antibodies against the SpTrf proteins as described (18) at a dilution of 1:100 in 100 μl staining medium for 30 min on ice followed by centrifugation at 400 × *g* for 5 min at 4°C and resuspension in staining medium. Goat anti-rabbit antibody conjugated to Alexa Fluor 488 (ThermoFisher Scientific) was employed as the secondary antibody and added at a dilution of 1:250 in 30 μl in staining medium followed by centrifugation at 400 × *g* for 5 min at 4°C and resuspension in staining medium. Negative controls omitted the primary antibodies to identify non-specific background by the secondary antibody. Cells were incubated with propidium iodide (PI; 1 μg/ml) to identify dead cells.

### Flow Cytometry

Coelomocytes were sorted on a FACSAria flow cytometer (BD Biosciences) equipped with blue, red, and violet lasers as described (45). Dead cell counts were gated out based on their nuclear PI excitation at 488 nm and detection with a 585/42 nm band pass (BP) filter. SpTrf^+^ small phagocytes were identified by Alexa Fluor 488 excitation at 488 nm and detection with a 530/30 nm BP filter. Red spherule cells were identified based on their natural auto-fluorescence in the far-red channel (660/20 nm BP filter) when excited at 633 nm. Flow cytometry data was analyzed with the FlowJo program Ver. 10 (FlowJo LLC).

### Single Cell Sorting and Whole Genome Amplification

SpTrf^+^ small phagocytes and red spherule cells were sorted as single cells into a 384-well plate containing 7 μl CMFSW-EH per well and observed by light and/or fluorescence microscopy to verify the presence of a single cell of the expected type in a well. After the verification of cell type per well, three SpTrf^+^ and three red spherule cells from each animal before challenge on day 0 and on days 1 and 2 after challenge were evaluated further. Cells were sorted into 4 μl D2 lysis buffer plus 3 μl 3.3X PBS of the REPLI-g Single Cell kit (Qiagen) and stored at −80°C until processing for WGA.

Sperm were collected from sea urchins after electric shock with a current of 16–20 mA for 1–2 min to induce spawning. Sperm (10^6^) cells were diluted into 1 ml of CMFSW-EH and stained with Hoechst 33342 (5 μg/ml) and PI (1 μg/ml) to determine the live/dead cell ratio. Stained cells were washed with the CMFSW-EH and diluted to a concentration of 0.25 cells/μl. Aliquots of 4 μl were placed in wells of a 384-well plate to achieve a statistical concentration of 0.1 cell per well. Wells with single Hoechst 33342^+^/PI^−^ sperm were identified by fluorescence microscopy and 4 μl D2 lysis buffer of the REPLI-g Single Cell kit (Qiagen) was added to each followed by WGA as described below.

### Amplification, Cloning, and Sequencing *SpTrf* Genes

WGA was carried out according to the manufacturer instructions (Qiagen) and products of the single cell samples were diluted 1:100 in water and employed as the template in PCR amplification with ExTaq™ DNA polymerase (Takara) using F2 and R9 degenerate primers (Table S1) according to Buckley and Smith (8) with a cycling program of 94°C for 3 min, 30 cycles of 94°C for 30 s, 55°C for 30 s, 72°C for 90 s, plus 72°C for 10 min. *SpGAPDH* (Genebank: XP_780116) was used as the gene amplification control with specific primers (Table S1) with the same cycling program. Amplicons were cloned into the pCR4-TOPO® TA vector (Invitrogen) and transformed into chemically competent TOP10® *E. coli* (Invitrogen). Bacteria were plated on Luria Burtani (LB) plates containing 100 μg/ml ampicillin and incubated at 37°C for 18 h. Colonies were picked, evaluated for relevant inserts (*SpTrf* or *SpGAPDH*) using specific primers, and grown in LB medium containing 100 μg/ml ampicillin at 37°C for 18 h. Plasmids were isolated with the QiaPrep® spin miniprep kit (Qiagen) and sequenced from both ends of the insert using T7 or T3 universal primers (for *SpTrf* ) or from one end using T7 primer (for *SpGAPDH*). Two *SpGAPDH* clones from a single coelomocyte (C1) and two from a sperm cell (S1) of the same genotype (animal #1) were sequenced. Only a single variation was identified among the sequences in one of the C1 clones (Figure S2). This variation may represent a PCR-induced error or a genuine allelic polymorphism.

### *SpTrf* Sequence Processing

Sequence processing was performed using Bioedit software (46) (http://www.mbio.ncsu.edu/bioedit/bioedit.html). Complementary sequences obtained from universal T7 and T3 sequencing primers were assembled by pairwise alignments and base calling mistakes were corrected upon inspection of the chromatograms to generate a consensus sequence and the vector sequences were trimmed. The element pattern in the second exon was identified by aligning the sequences manually with the set of *SpTrf* genes reported previously (8). Sequences were categorized first according to element patterns of the second exon and next according to sequence variants of these categories. Sequences were named based on animal number, element pattern, and sequence variant number (Table S2).

### *SpTrf* Sequence Diversity

The consensus insert sequences for all samples based on reads obtained from T3 and T7 sequencing primers were first translated to amino acid sequences and used in a multiple global alignment (ClustalW) performed in Bioedit software (46) using standard parameters. The amino acid alignment from ClustalW was optimized manually and reverted to nucleotides for subsequent analysis. Diversity (entropy) was calculated for each nucleotide/amino acid position using the equation: diversity = –freq × ln(freq). The episodic diversifying selection for the *SpTrf* sequences derived from single cells was conducted using MEME (https://www.datamonkey.org/meme) (47).

### Quantitative PCR

qPCR (10 μl) was performed using 1 μl of 1:100 dilution of WGA single cell genomic DNA or 1–5 ng of purified genomic DNA from ~10^6^ coelomocytes. *SpTrf* gene copy number analysis for single cells was performed with the ΔΔCT method relative to the *SpGAPDH* control gene and normalized against the sperm values for the same animal. All experiments were performed using the CFX96 Real-Time PCR System and the iTaq Universal SYBR Green Supermix (BioRad). The BioRad CFX Manager software was employed for all analyses. All primers were validated prior to use (Table S1). All qPCR amplicons were sequenced and verified as *SpTrf* sequences.

### Phylogenetic Analysis

Phylogenetic trees were generated based on the manually corrected ClustalW alignment with Geneious software Ver. 6.1.7 (Biomatters Ltd.) using PhyML and PAUP plugins with maximum parsimony, neighbor joining, and maximum likelihood approaches with different genetic models. All trees gave similar results. The maximum likelihood was chosen as the example of a tree to illustrate *SpTrf* phylogeny and was obtained by PhyML with Jukes-Cantor genetic distance models with 1,000 bootstrap replicates.

## Data Availability

The datasets generated for this study can be found in Nucleotide database, GenBank, NCBI, KY774859-KY774912.

## Author Contributions

MO, BR, and LCS conceived and designed the experiments. MO, BR, TH, JA, G-YK, and CR performed the experiments. MO, TH, LG, BR, and JA analyzed the data. TH, MO, LG, LCS, and BR contributed reagents, materials and research tools. MO and LCS wrote the paper.

### Conflict of Interest Statement

The authors declare that the research was conducted in the absence of any commercial or financial relationships that could be construed as a potential conflict of interest.
