# Supplementary material for: Individual Sea Urchin Coelomocytes Undergo Somatic Immune Gene Diversification
Source: Front Immunol. 2019 Jun 6;10:1298. doi: 10.3389/fimmu.2019.01298 (PMC6563789; doi:10.3389/fimmu.2019.01298)
Supplement: Supplementary file 1 [file Table_1.DOCX]

Individual sea urchin coelomocytes undergo somatic immune gene diversification

Matan Oren, Benyamin Rosental, Teresa S. Hawley, Gi-Young Kim, Jacob Agronin, Caroline R. Reynolds, Leon Grayfer, L. Courtney Smith

**Supplementary tables**

Table S1. Primers used for sequencing and qPCR

| Primer type | Primer set | Source tissue | Sequence |
| --- | --- | --- | --- |
| Degenerate *SpTrf* primers | F2  R9 | All | F AAGMGATTWCAATGAACKRCGAG  R CTTHARGTGGTGAARATGTCG |
| SpGAPD control | SpGAPDH-long | All | F GATCGATGGCAACTGCTGTTCC  R TTTCCGCGTTATCGACCTCATC |
| qPCR primers | SpGAPDH | All | F GTGTATTCACAACCATCGACAAG  R CCTCGTTGACACCCATAACA |
|  | Animal 1 -all | S1-2, C1-2 | F GATCTCCCAGGCGTGATG  R GATGCTCCTTCTCCTCACTTT |
|  | G2-1 | S1-2 | F GACAAATTGGTGATGGTCTAGGA  R AGGAGGATTGCCGAAGAAAG |
|  | D1-1 | S1-2, C1-2 | F CACCACCACCGTCATCATAA  R GTACTGGAAGGGCCTTGTATC |
|  | B3-1-7 | S1-2, C1-2 | F CACCACCACCGTCATCATAA  R GGAAGGGCCTCGTATCATTT |
|  | E2-1-5 | S1-2, C1-2 | F GGACCGACCAGAGGAACAA  R TGGTGGCGAGGGTGAG |
|  | 01-1 | S1-2, C1-2 | F GGTGATGGAGAAGAAGAAACTGATG  R TACCATGATGACGACGTCCAG |
|  | Animal 2 -all | S2-2, C2-2 | F GCTCCAGACCAGATGGT  R CCTCCTAGACCATCACCAAT |
|  | A3-1 | S2-2, C2-2 | F CAAGGTGCAGGAAGACCTTT  R GTGGCGATCGTGGTGAC |
|  | D1-5-8 | S2-2, C2-2 | F CCCGGAGATGGATGGAC  R AGCACCATCTGGTCTGG |
|  | C3-1-3 | S2-2, C2-2 | F TCGCTACCACCATCATCATAAC  R CCGGAAGGGTCTTGTATCAAT |
|  | E2 6-11 | S2-2, C2-2 | F GGATCGACCAGAGGAACAAC  R TGGTTACGATGATGGTGGTG |
|  | 01-2 | S2-2 | F GTCGTCATCATGGTAGAAAGCC  R GGTGGTCTCCCGTCTCATTA |
|  | Animal 3 -all | S3-3, C3-4 | F GATGGTGCTGGAGGAAGAC  R GGGCAGCATCAGTTTCTTCT |
|  | A4-1 | S3-3 | F CAAGGTGCAGGAAGACCTTT  R GTGGCGATCGTGGTGAC |
|  | D1-9-12 | S3-3 | F AGGTTCGATGGACCTGACT  R GAACCTCCTTCCACCCATTG |
|  | E2 12-15 | S3-3, C3-4 | F GACCGACCAGAGGAACAA  R GATGATGATGCTGGTGGTG |
|  | 02-1-4 | S3-3, C3-4 | F CAACCATACAGAAGGTCACCAG  R ATCGGTCTGTCCTCTCCATC |

Table S2. Cloned and sequenced *SpTrf* gene amplicons from sea urchins 1-3.

| Accession number | Gene variant | Tissue/animal number-cell^a^ | Amplicon length (nt)^b^ | # of clones^c^ | Gene copy ratio; C vs. S^d^ |
| --- | --- | --- | --- | --- | --- |
| Animal 1 | | | | | |
| KY774859 | *G2*-1 | S1-2 | 1364 | 1 | 0 |
| KY774863 | *A5*-1 | S1-2 | 1262 | 2 | - |
| KY774866 | *D1*-1 | S1-2, C1-2 | 1022 | 5, 3 | 0.1 |
| KY774867 | *D1*-2 | S1-2 | 1022 | 2 |  |
| KY774868 | *D1*-3 | S1-2 | 1022 | 2 |  |
| KY774869 | *D1*-4 | S1-2 | 1022 | 1 |  |
| KY774883 | *D5*-1 | S1-2 | 947 | 2 | - |
| KY774886 | *B3*-1 | S1-2, C1-2 | 926 | 3, 4 | 0.17 |
| KY774887 | *B3*-2 | S1-2 | 926 | 2 |  |
| KY774888 | *B3*-3 | S1-2 | 926 | 1 |  |
| KY774889 | *B3*-4 | S1-2 | 926 | 2 |  |
| KY774890 | *B3*-5 | S1-2 | 926 | 2 |  |
| KY774892 | *B3*-6 | C1-2 | 926 | 3 |  |
| KY774894 | *E2*-1 | S1-2, C1-2 | 812 | 5, 4 | 0.41 |
| KY774896 | *E2*-2 | S1-2 | 812 | 2 |  |
| KY774897 | *E2*-3 | S1-2 | 812 | 1 |  |
| KY774898 | *E2*-4 | C1-2 | 812 | 10 |  |
| KY774899 | *E2*-5 | C1-2 | 812 | 4 |  |
| KY774910 | *01*-1 | S1-2, C1-2 | 722 | 7, 9 | 0.12 |
| Animal 2 | | | | | |
| KY774860 | *A3*-1 | S2-2, C2-2 | 1358 | 3, 1 | 0.44 |
| KY774864 | *C2*-1 | S2-2 | 1028 | 3 | - |
| KY774871 | *D1*-5 | S2-2 | 1022 | 2 | 0.46 |
| KY774872 | *D1*-6 | S2-2 | 1022 | 3 |  |
| KY774873 | *D1*-7 | C2-2 | 1022 | 2 |  |
| KY774874 | *D1*-8 | C2-2 | 1022 | 1 |  |
| KY774879 | *C3*-1 | S2-2 | 953 | 6 | 2.35 |
| KY774880 | *C3*-2 | C2-2 | 953 | 6 |  |
| KY774881 | *C3*-3 | C2-2 | 953 | 11 |  |
| KY774884 | *D5*-2 | S2-2 | 947 | 3 | - |
| KY774893 | *B3*-7 | S2-2 | 926 | 3 | - |
| KY774900 | *E2*-6 | S2-2 | 812 | 3 | 0.66 |
| KY774901 | *E2*-7 | S2-2 | 812 | 3 |  |
| KY774902 | *E2*-8 | S2-2 | 812 | 2 |  |
| KY774903 | *E2*-9 | C2-2 | 812 | 2 |  |
| KY774904 | *E2*-10 | C2-2 | 812 | 1 |  |
| KY774905 | *E2*-11 | C2-2 | 812 | 6 |  |
| KY774912 | *01*-2 | S2-2 | 722 | 3 | 0 |

| Animal 3 | | | | | |
| --- | --- | --- | --- | --- | --- |
| KY774862 | *A4*-1 | S3-3 | 1343 | 3 | 0 |
| KY774865 | *C2*-2 | S3-3 | 1028 | 4 | - |
| KY774875 | *D1*-9 | S3-3 | 1022 | 4 | 0 |
| KY774876 | *D1*-10 | S3-3 | 1022 | 4 |  |
| KY774877 | *D1*-11 | S3-3 | 1022 | 2 |  |
| KY774878 | *D1*-12 | S3-3 | 1022 | 2 |  |
| KY774882 | *C3*-4 | S3-3 | 953 | 3 | - |
| KY774885 | *D5*-3 | S3-3 | 947 | 3 | - |
| KY774906 | *E2*-12 | S3-3 | 812 | 3 | 5.96 |
| KY774907 | *E2*-13 | C3-4 | 812 | 16 |  |
| KY774908 | *E2*-14 | C3-4 | 812 | 2 |  |
| KY774909 | *E2*-15 | C3-4 | 812 | 2 |  |
| KY774913 | *01*-3 | S3-3 | 722 | 5 | - |
| KY774915 | *02*-1 | S3-3, C3-4 | 719 | 3, 23 | 1.47 |
| KY774917 | *02*-2 | S3-3 | 719 | 1 |  |
| KY774918 | *02*-3 | S3-3 | 719 | 2 |  |
| KY774919 | *02*-4 | S3-3 | 719 | 2 |  |

^a^Tissue is coelomocytes (C) or sperm (S). Animals are 1-3 as in figure 4b in the main paper. Cell numbers are 1-4, which are circled in figure 4b in the main paper.

^b^The amplicon length in nucleotides includes primers.

^c^The number of transformed *E. coli* colonies that were picked randomly and processed for sequencing the *SpTrf* insert of the TOPO-TA4 vector (Thermo Fisher).

^d^qPCR was carried out using the WGA products from the single cells with primers designed to amplify specifically one or more genes of the selected element patterns and normalized to *SpGAPDH* (Table S1). Results were used to estimate gene copy in coelomocytes relative to sperm. Genes that were not tested with qPCR are marked with “-“.
