# Supplementary material for: Individual Sea Urchin Coelomocytes Undergo Somatic Immune Gene Diversification
Source: Front Immunol. 2019 Jun 6;10:1298. doi: 10.3389/fimmu.2019.01298 (PMC6563789; doi:10.3389/fimmu.2019.01298)
Supplement: Supplementary file 2 [file Data_Sheet_1.PDF]

Individual sea urchin coelomocytes undergo somatic immune gene diversification

Matan Oren, Benyamin Rosental, Teresa S. Hawley, Gi-Young Kim, Jacob Agronin, Caroline R. Reynolds, Leon Grayfer, L. Courtney Smith

Supplementary Figures

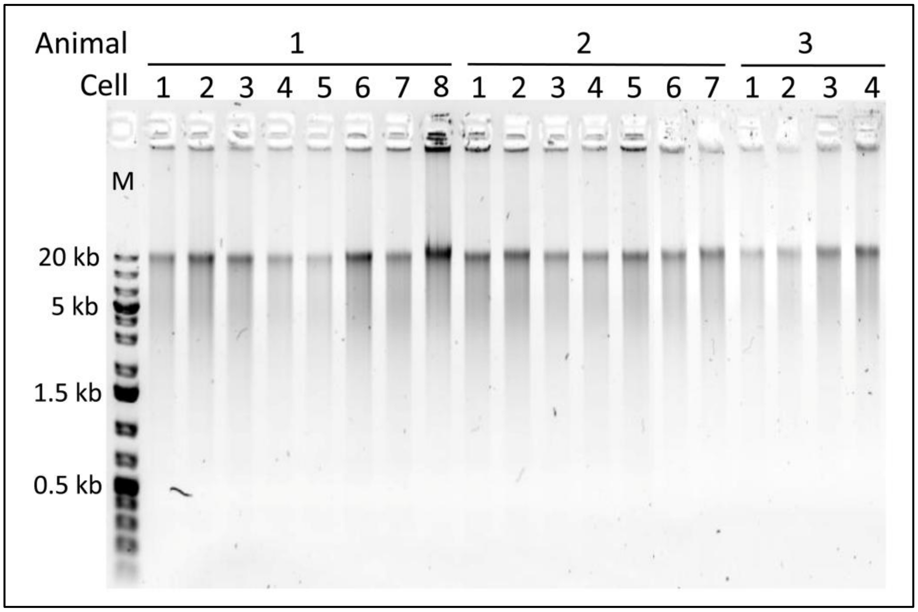

Figure S1. Products of whole genome amplification of single coelomocytes. The major amplification signal is ≥ 20 kb with a smear of smaller sizes. M indicates the DNA size marker.

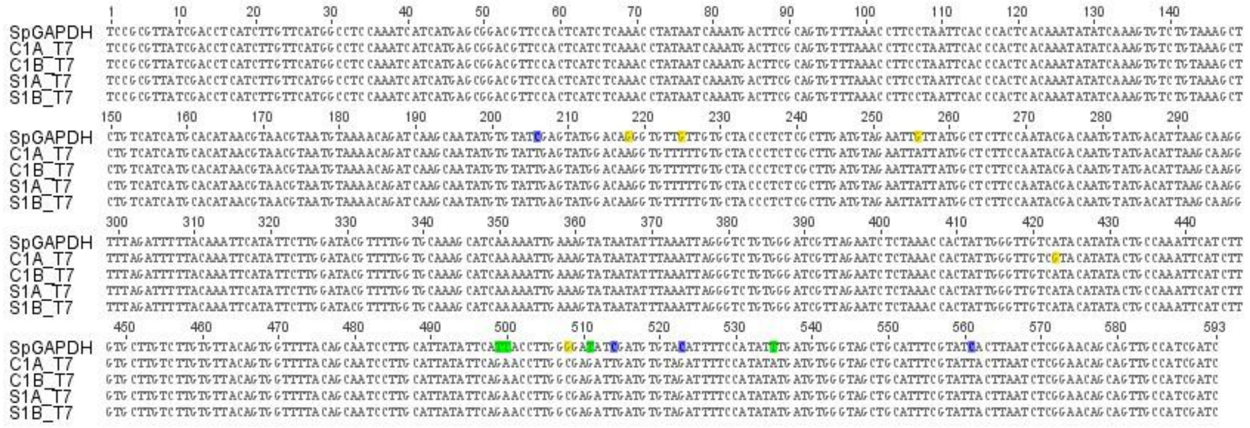

Figure S2. *SpGAPDH* amplicon sequence alignment. The top sequence is the relevant part of *SpGAPDH* genebank sequence (ref. number: XM_775023.4). Two clones of each C1 and S1 amplicons were sequenced (A and B). While amplified sequences showed mild sequence difference (in 12 nt positions) compared with genebank sequence, only one nucleotide mismatch was identified among them (position 424 of C1B amplicon). This single missmatch may represent a real SNP in a second allele of C1 genome or it may be an error caused by the polymerase used in the PCR reaction.

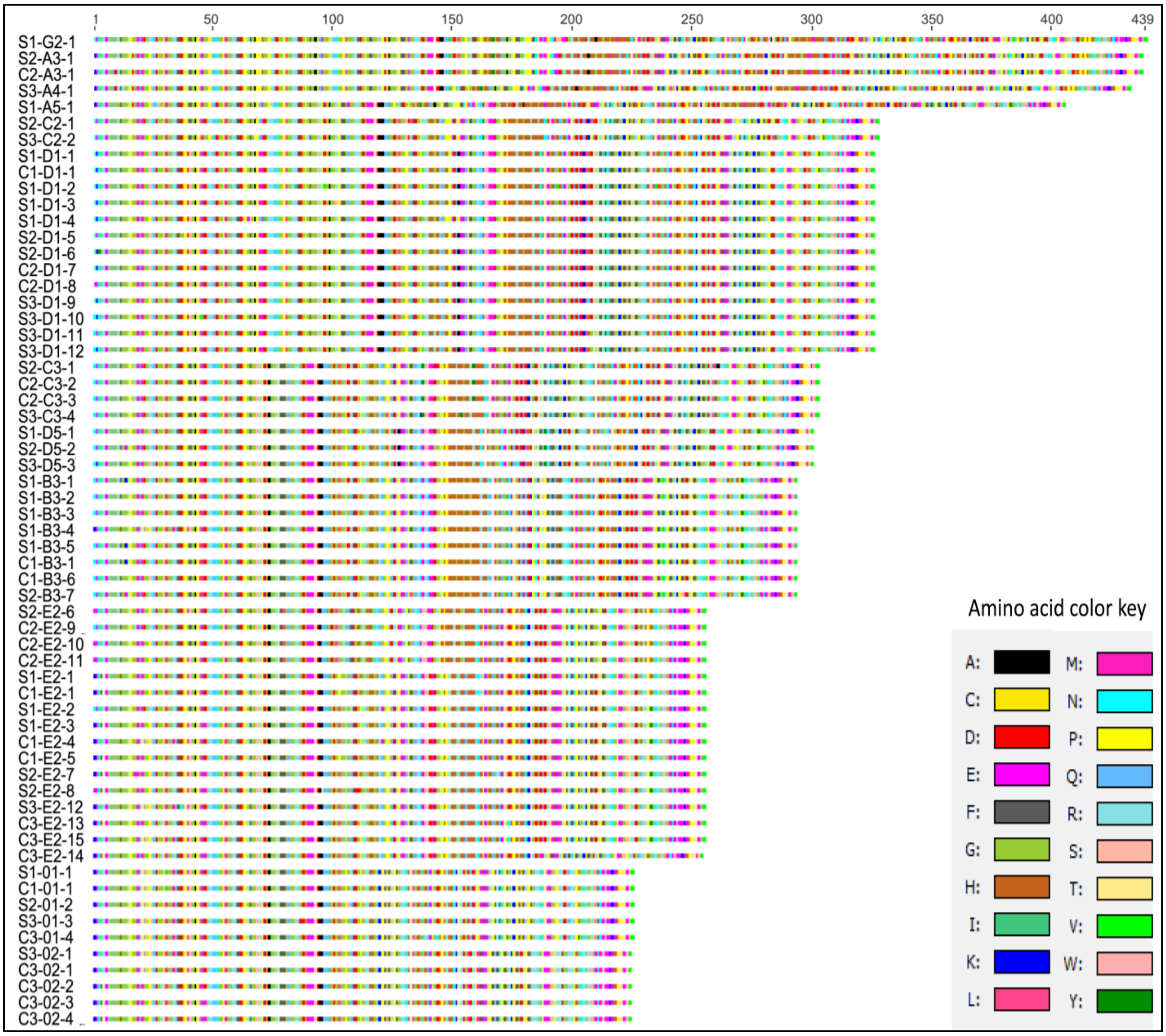

Figure S3. Amino acid sequence of the *SpTrf* ORFs from single cells. All sequences were translated into complete protein sequences with no missense or early stop codons. Different *SpTrf* sequence lengths are correlated to the element patterns identified.

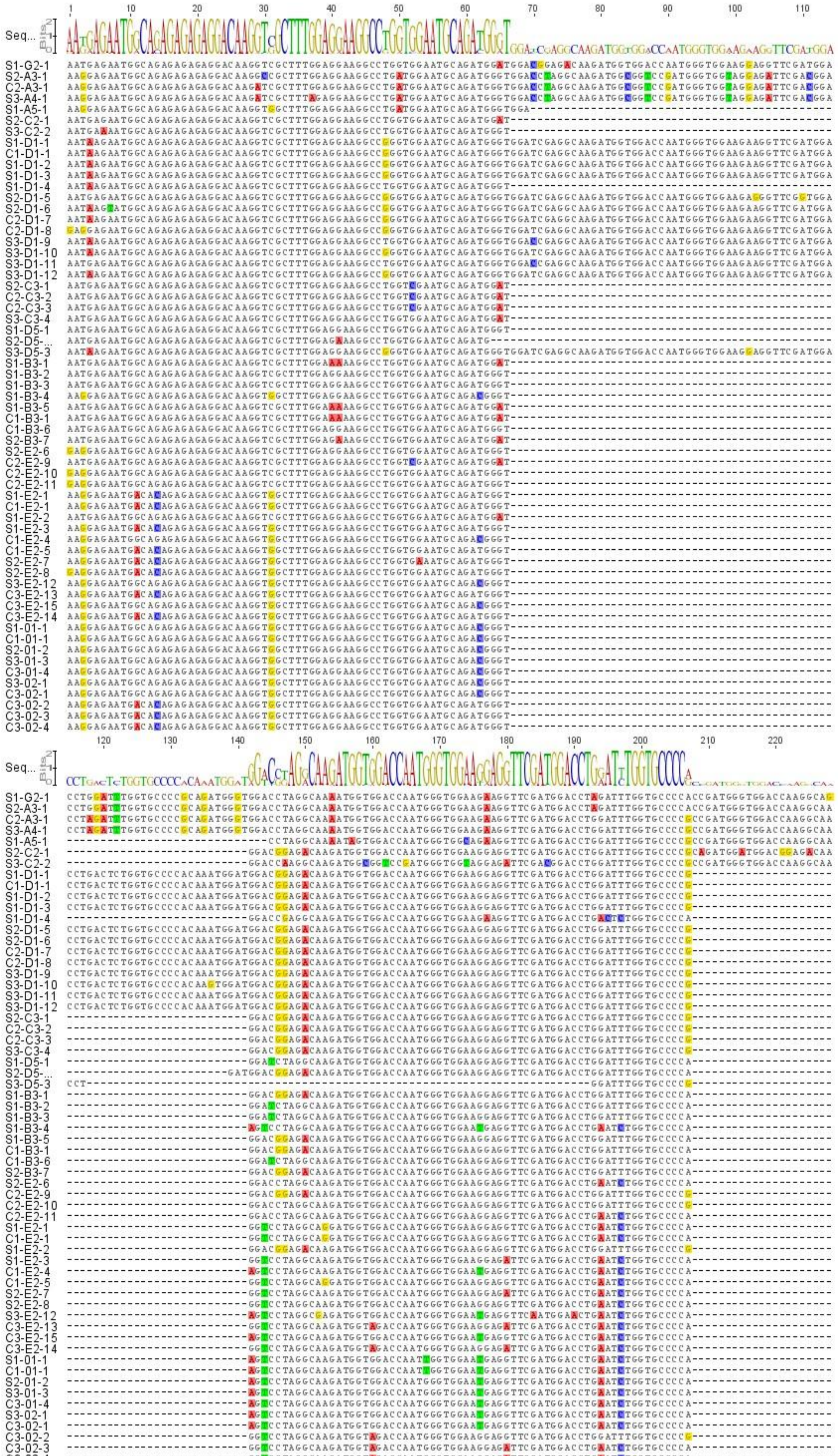
Seq...
Bits
S1-G2-1
S2-A3-1
C2-A3-1
S3-A4-1
S1-A5-1
S2-C2-1
S3-C2-2
S1-D1-1
C1-D1-1
S1-D1-2
S1-D1-3
S1-D1-4
S2-D1-5
S2-D1-6
C2-D1-7
C2-D1-8
S3-D1-9
S3-D1-10
S3-D1-11
S3-D1-12
S2-C3-1
C2-C3-2
C2-C3-3
S3-C3-4
S1-D5-1
S2-D5-...
S3-D5-3
S1-B3-1
S1-B3-2
S1-B3-3
S1-B3-4
S1-B3-5
C1-B3-1
C1-B3-6
S2-B3-7
S2-E2-6
C2-E2-9
C2-E2-10
C2-E2-11
S1-E2-1
C1-E2-1
S1-E2-2
S1-E2-3
C1-E2-4
C1-E2-5
S2-E2-7
S2-E2-8
S3-E2-12
C3-E2-13
C3-E2-15
C3-E2-14
S1-01-1
C1-01-1
S2-01-2
S3-01-3
C3-01-4
S3-02-1
C3-02-1
C3-02-2
C3-02-3
C3-02-4

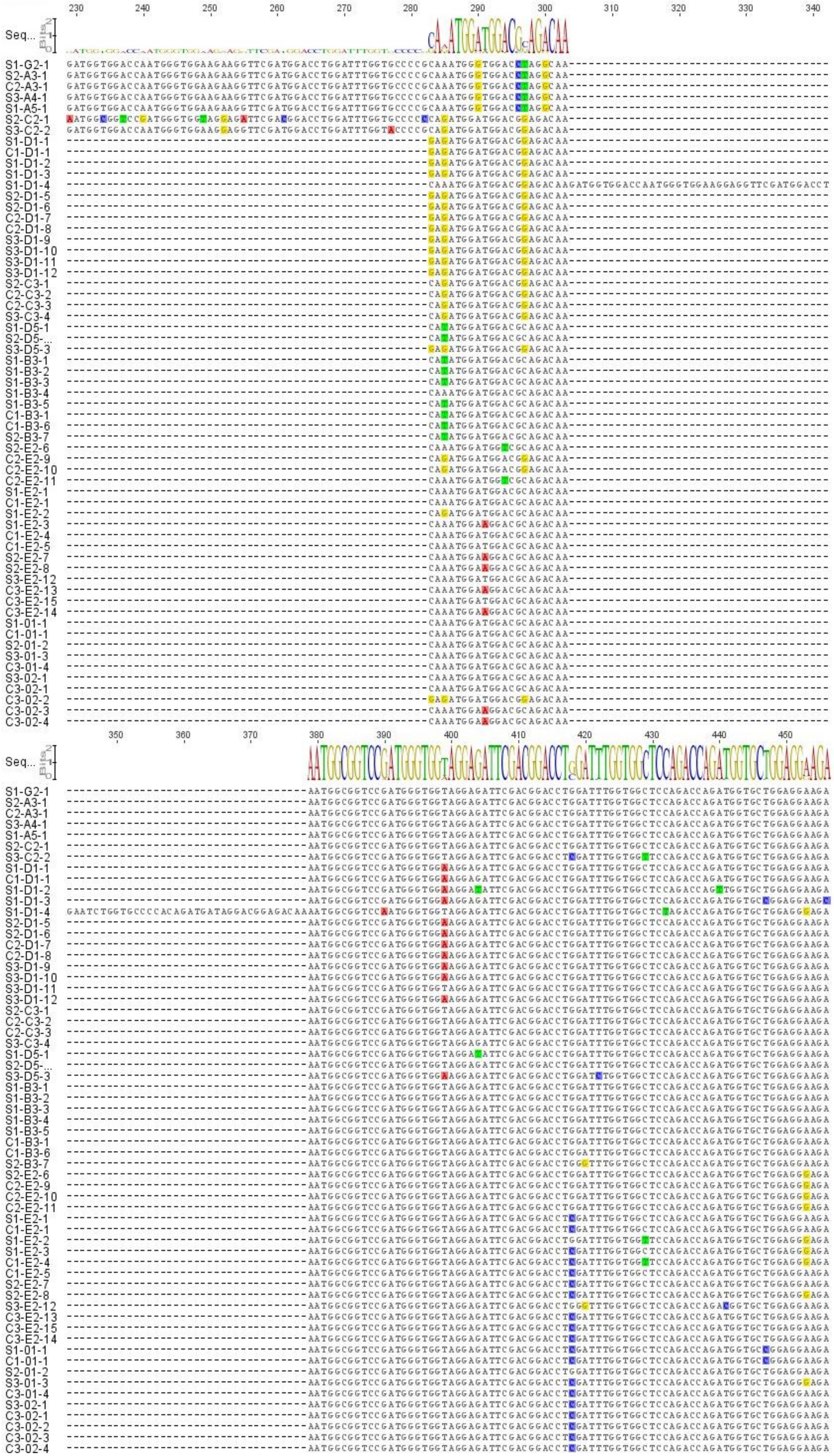
230 240 250 260 270 280 290 300 310 320 330 340
Seq...
Bits
S1-G2-1
S2-A3-1
C2-A3-1
S3-A4-1
S1-A5-1
S2-C2-1
S3-C2-2
S1-D1-1
C1-D1-1
S1-D1-2
S1-D1-3
S1-D1-4
S2-D1-5
S2-D1-6
C2-D1-7
C2-D1-8
S3-D1-9
S3-D1-10
S3-D1-11
S3-D1-12
S2-C3-1
C2-C3-2
C2-C3-3
S3-C3-4
S1-D5-1
S2-D5-...
S3-D5-3
S1-B3-1
S1-B3-2
S1-B3-3
S1-B3-4
S1-B3-5
C1-B3-1
C1-B3-6
S2-B3-7
S2-E2-6
C2-E2-9
C2-E2-10
C2-E2-11
S1-E2-1
C1-E2-1
S1-E2-2
S1-E2-3
C1-E2-4
C1-E2-5
S2-E2-7
S2-E2-8
S3-E2-12
C3-E2-13
C3-E2-15
C3-E2-14
S1-01-1
C1-01-1
S2-01-2
S3-01-3
C3-01-4
S3-02-1
C3-02-1
C3-02-2
C3-02-3
C3-02-4
350 360 370 380 390 400 410 420 430 440 450
Seq...
Bits

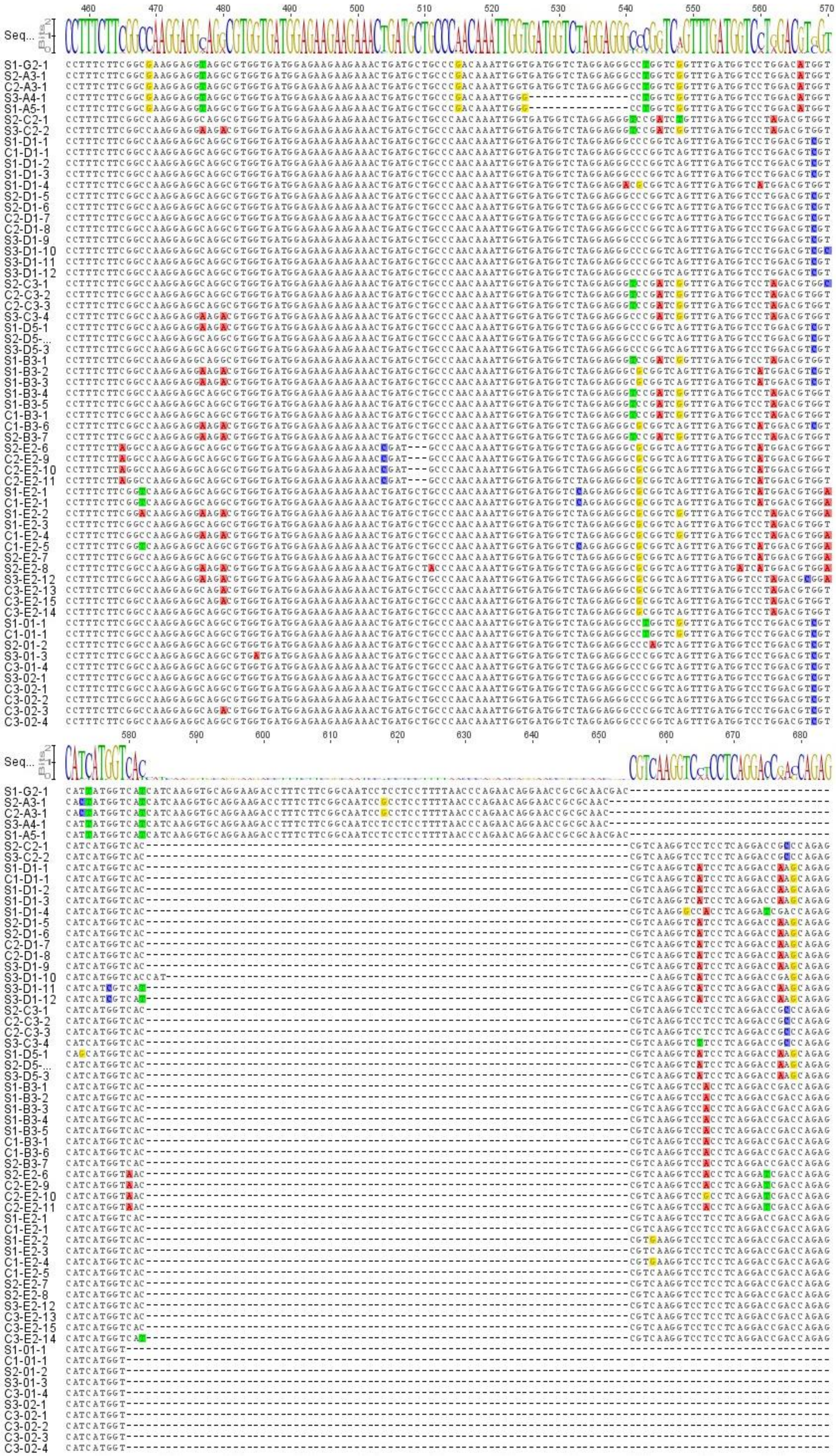

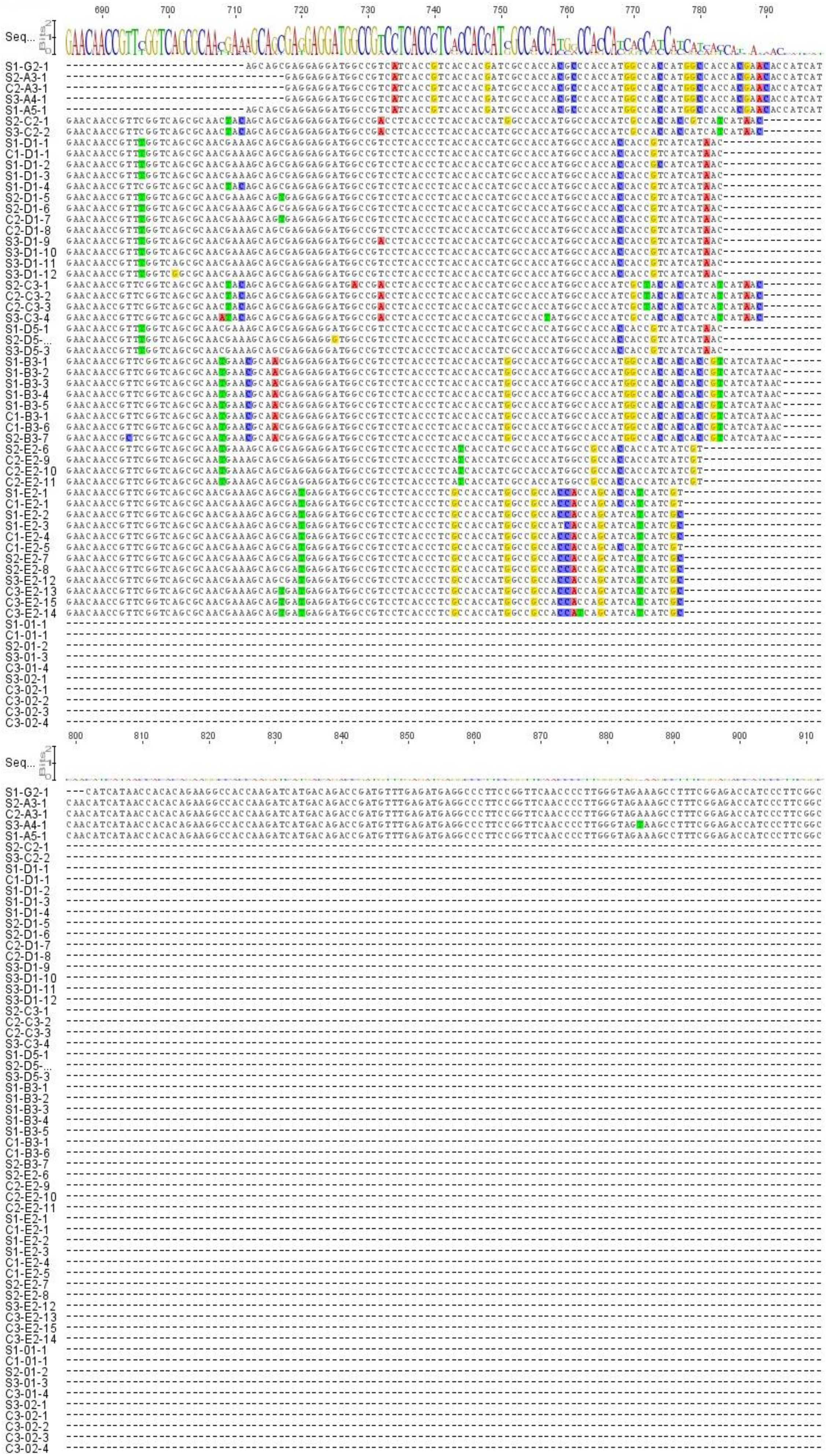
690 700 710 720 730 740 750 760 770 780 790
Seq...
Bits
S1-G2-1
S2-A3-1
C2-A3-1
S3-A4-1
S1-A5-1
S2-C2-1
S3-C2-2
S1-D1-1
C1-D1-1
S1-D1-2
S1-D1-3
S1-D1-4
S2-D1-5
S2-D1-6
C2-D1-7
C2-D1-8
S3-D1-9
S3-D1-10
S3-D1-11
S3-D1-12
S2-C3-1
C2-C3-2
C2-C3-3
S3-C3-4
S1-D5-1
S2-D5-...
S3-D5-3
S1-B3-1
S1-B3-2
S1-B3-3
S1-B3-4
S1-B3-5
C1-B3-1
C1-B3-6
S2-B3-7
S2-E2-6
C2-E2-9
C2-E2-10
C2-E2-11
S1-E2-1
C1-E2-1
S1-E2-2
S1-E2-3
C1-E2-4
C1-E2-5
S2-E2-7
S2-E2-8
S3-E2-12
C3-E2-13
C3-E2-15
C3-E2-14
S1-01-1
C1-01-1
S2-01-2
S3-01-3
C3-01-4
S3-02-1
C3-02-1
C3-02-2
C3-02-3
C3-02-4
800 810 820 830 840 850 860 870 880 890 900 910

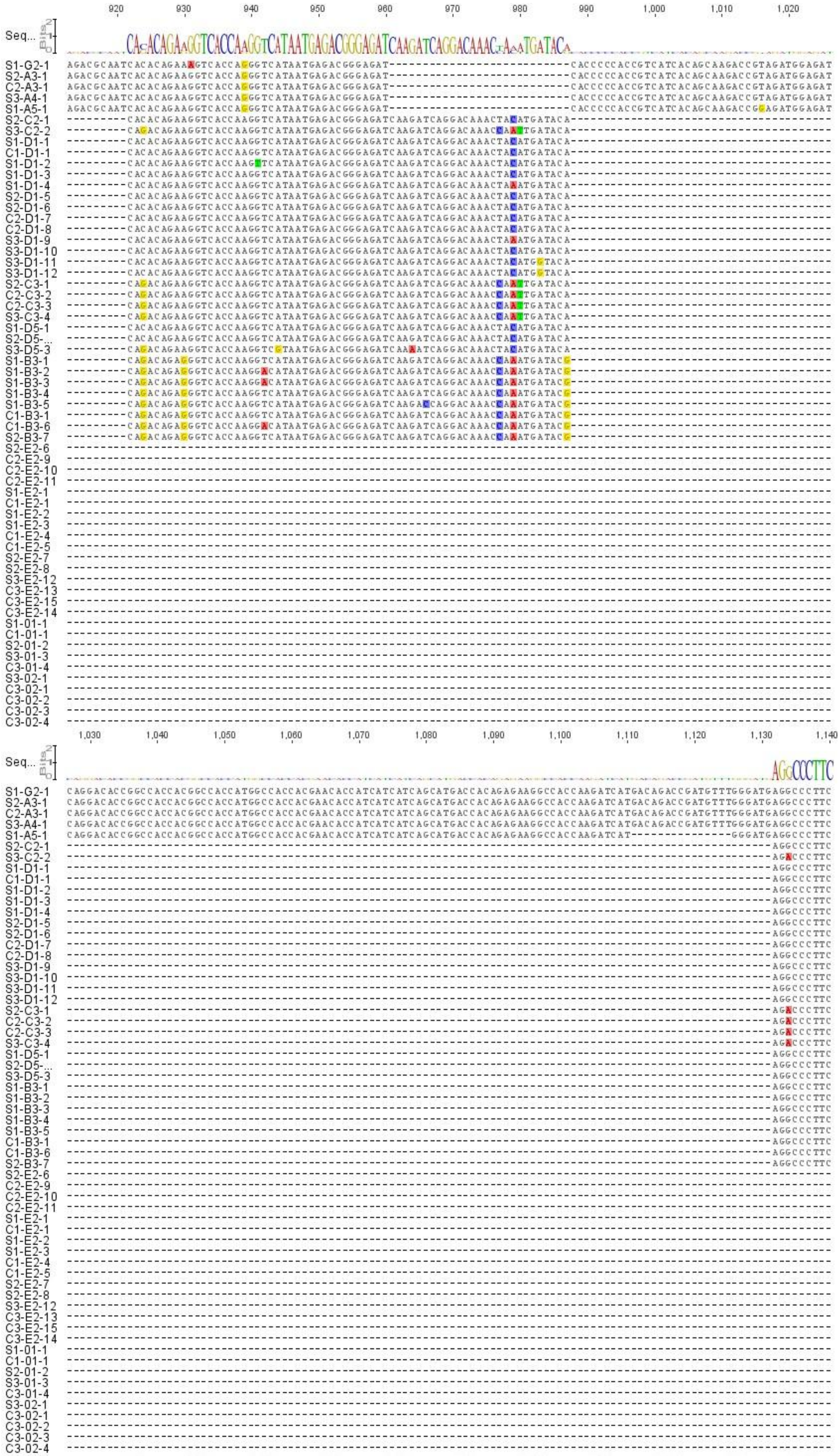

920 930 940 950 960 970 980 990 1,000 1,010 1,020
Seq...
S1-G2-1
S2-A3-1
C2-A3-1
S3-A4-1
S1-A5-1
S2-C2-1
S3-C2-2
S1-D1-1
C1-D1-1
S1-D1-2
S1-D1-3
S1-D1-4
S2-D1-5
S2-D1-6
C2-D1-7
C2-D1-8
S3-D1-9
S3-D1-10
S3-D1-11
S3-D1-12
S2-C3-1
C2-C3-2
C2-C3-3
S3-C3-4
S1-D5-1
S2-D5-...
S3-D5-3
S1-B3-1
S1-B3-2
S1-B3-3
S1-B3-4
S1-B3-5
C1-B3-1
C1-B3-6
S2-B3-7
S2-E2-6
C2-E2-9
C2-E2-10
C2-E2-11
S1-E2-1
C1-E2-1
S1-E2-2
S1-E2-3
C1-E2-4
C1-E2-5
S2-E2-7
S2-E2-8
S3-E2-12
C3-E2-13
C3-E2-15
C3-E2-14
S1-01-1
C1-01-1
S2-01-2
S3-01-3
C3-01-4
S3-02-1
C3-02-1
C3-02-2
C3-02-3
C3-02-4
1,030 1,040 1,050 1,060 1,070 1,080 1,090 1,100 1,110 1,120 1,130 1,140

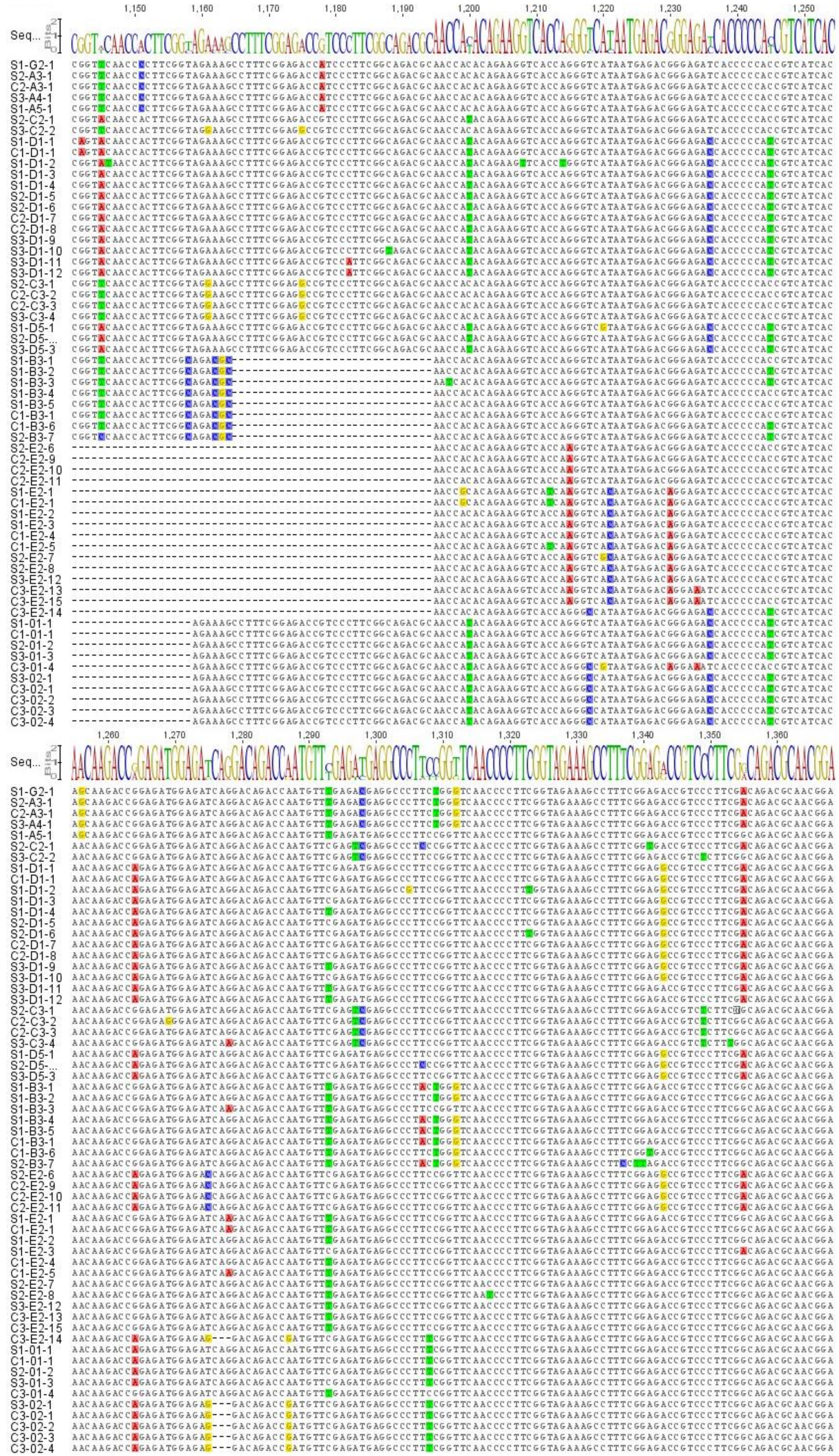
1,150 1,160 1,170 1,180 1,190 1,200 1,210 1,220 1,230 1,240 1,250
Seq...
Bits
S1-G2-1
S2-A3-1
C2-A3-1
S3-A4-1
S1-A5-1
S2-C2-1
S3-C2-2
S1-D1-1
C1-D1-1
S1-D1-2
S1-D1-3
S1-D1-4
S2-D1-5
S2-D1-6
C2-D1-7
C2-D1-8
S3-D1-9
S3-D1-10
S3-D1-11
S3-D1-12
S2-C3-1
C2-C3-2
C2-C3-3
S3-C3-4
S1-D5-1
S2-D5-...
S3-D5-3
S1-B3-1
S1-B3-2
S1-B3-3
S1-B3-4
S1-B3-5
C1-B3-1
C1-B3-6
S2-B3-7
S2-E2-6
C2-E2-9
C2-E2-10
C2-E2-11
S1-E2-1
C1-E2-1
S1-E2-2
S1-E2-3
C1-E2-4
C1-E2-5
S2-E2-7
S2-E2-8
S3-E2-12
C3-E2-13
C3-E2-15
C3-E2-14
S1-01-1
C1-01-1
S2-01-2
S3-01-3
C3-01-4
S3-02-1
C3-02-1
C3-02-2
C3-02-3
C3-02-4
1,260 1,270 1,280 1,290 1,300 1,310 1,320 1,330 1,340 1,350 1,360

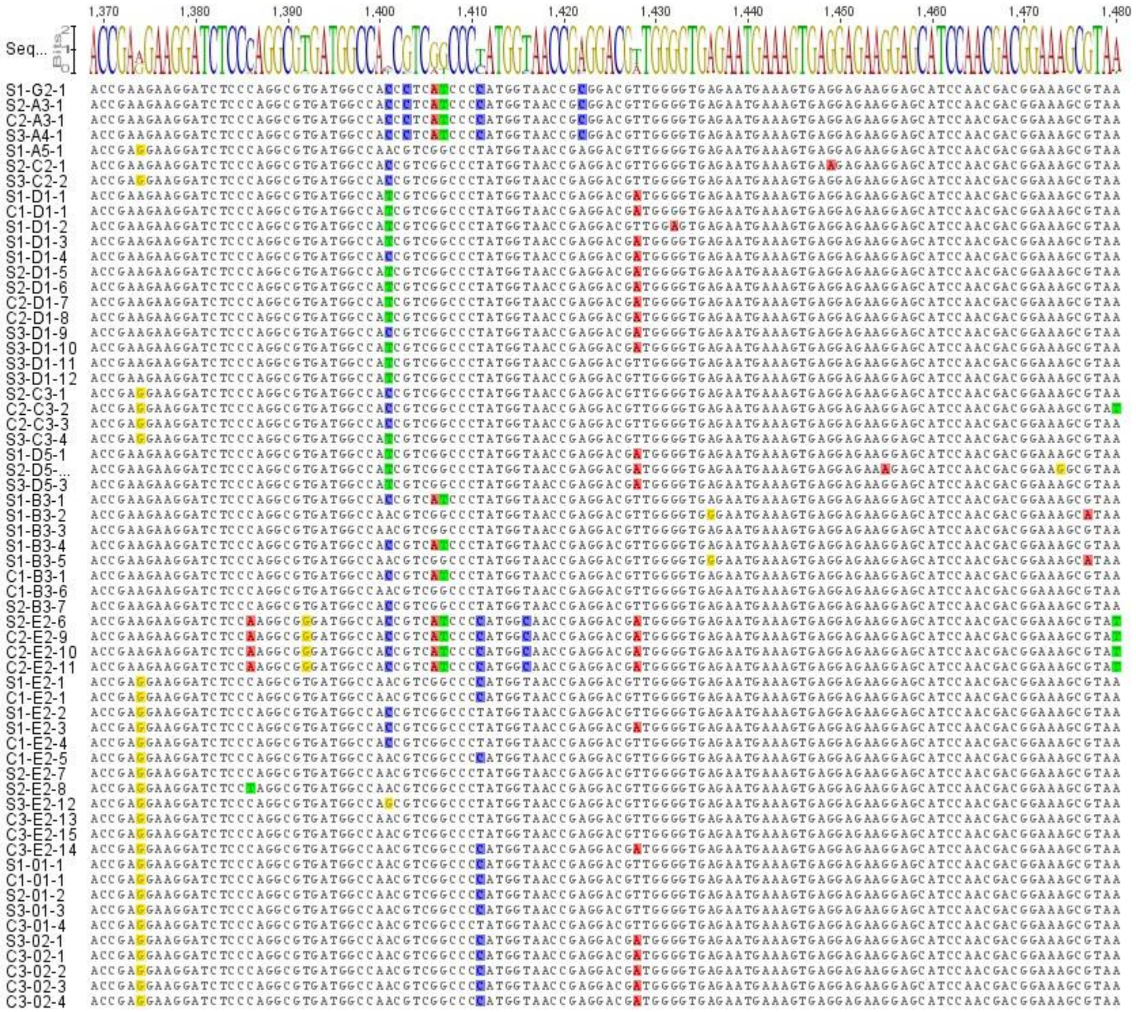

Figure S4. Detailed alignment of *SpTrf* sequences from individual sea urchin coelomocytes. Sequence logo is presented on top. SNPs are highlighted, gaps are marked with '-'

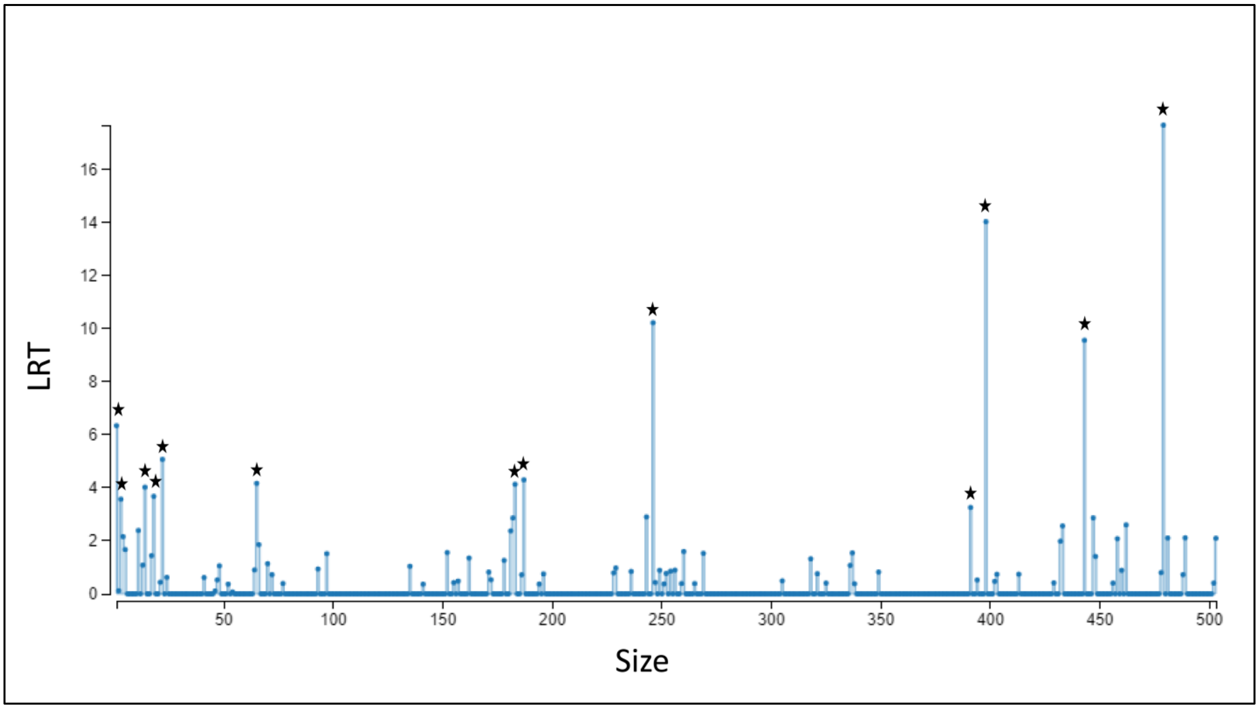

Figure S5. Positions identified for positive selection for diversification of *SpTrf* genes from single cells are spread throughout the amplicon. Likelihood ratio test (LRT) for episodic diversification in the *SpTrf* amplicon sequence alignment is shown for each nucleotide position. The X axis indicates the amino acid positions. Thirteen positions (indicated by black stars) are likely positively diversified with *p* value < 0.1.
